# A review of methodology and analysis of nutrition and mortality surveys conducted in humanitarian emergencies from October 1993 to April 2004

**DOI:** 10.1186/1742-7622-4-10

**Published:** 2007-06-01

**Authors:** Claudine Prudhon, Paul B Spiegel

**Affiliations:** 1 United Nations Standing Committee on Nutrition, c/o World Health Organization, 20 Avenue Appia, CH 1211, Geneva 27, Switzerland; 2 United Nations High Commissioner for Refugees, PO Box 2500, CH 1211, Genève 2 Dépôt, Switzerland

## Abstract

**Background:**

Malnutrition prevalence and mortality rates are increasingly used as essential indicators to assess the severity of a crisis, to follow trends, and to guide decision-making, including allocation of funds. Although consensus has slowly developed on the methodology to accurately measure these indicators, errors in the application of the survey methodology and analysis have persisted. The aim of this study was to identify common methodological weaknesses in nutrition and mortality surveys and to provide practical recommendations for improvement.

**Methods:**

Nutrition (N = 368) and crude mortality rate (CMR; N = 158) surveys conducted by 33 non-governmental organisations and United Nations agencies in 17 countries from October 1993 to April 2004 were analysed for sampling validity, precision, quality of measurement and calculation according to several criteria.

**Results:**

One hundred and thirty (35.3%) nutrition surveys and 5 (3.2%) CMR surveys met the criteria for quality. Quality of surveys varied significantly depending on the agency. The proportion of nutrition surveys that met criteria for quality rose significantly from 1993 to 2004; there was no improvement for mortality surveys during this period.

**Conclusion:**

Significant errors and imprecision in the methodology and reporting of nutrition and mortality surveys were identified. While there was an improvement in the quality of nutrition surveys over the years, the quality of mortality surveys remained poor. Recent initiatives aimed at standardising nutrition and mortality survey quality should be strengthened. There are still a number of methodological issues in nutrition and mortality surveys in humanitarian emergencies that need further study.

## Background

Humanitarian emergencies increased five-fold in the last decade of the twentieth century [1]. By the end of 2005, there were an estimated 23.7 million internally displaced persons and approximately 8.3 million refugees [2,3]; in 2005, an estimated 157.5 million people were affected by natural disasters [4]. Concomitant with the increase in humanitarian emergencies and the consequent increase in morbidity and mortality among the affected populations, the discipline of emergency public health and nutrition has evolved, including efforts to improve assessments and monitoring of health and nutrition situations [5].

Nutritional status and mortality rates are now widely used as essential indicators to assess the degree of severity of a crisis, to follow trends, and to guide decision making, which includes the allocation of funds [6,7]. The most widely accepted indicator for measuring the prevalence of acute malnutrition is the weight-for-height index, expressed as a Z-score, with the presence of oedema [8,9]. In acute humanitarian emergencies, mortality rates are generally expressed as number of deaths per 10,000 people per day [6]. Cross-sectional surveys using cluster or systematic sampling are commonly used to assess these indicators during or immediately after a humanitarian emergency [8,9]. Adequate sampling methodology and sample size are essential to ensure the representativeness and accuracy of a survey as well as the precision of the results, respectively. For acute malnutrition, there is almost consensus on the survey methodology, anthropometric measurements, calculation of nutrition indices and statistical description of the prevalence among children between six and 59 months in humanitarian emergencies [5,10]. Furthermore, agreement is slowly developing on methods to accurately measure mortality in humanitarian emergencies using cross-sectional surveys [7,11]. However, errors in the application of these survey methodologies in the field persist. Studies conducted in Somalia, Ethiopia and Iraq showed the lack of rigour in many nutrition [12-14] and mortality surveys [12].

The system on Nutrition Information in Crisis Situations (formerly Refugee Nutrition Information System) of the United Nations Standing Committee on Nutrition (UN/SCN) was established in 1993 to collect and disseminate nutrition information through quarterly reports. Nearly 1,000 nutrition survey reports, some of them including mortality surveys, have been received from non-governmental organisations (NGOs) and UN agencies since the establishment of the system; it represents the largest and most varied collection of such surveys in the world. In this article, we review the quality of the methodology used in these surveys and examine the trends in quality from 1993 to early 2004. The objectives of this paper are to identify common methodological errors in nutrition and mortality surveys conducted in humanitarian emergencies, to examine the trends over time, and to provide recommendations on how to improve surveys in the future.

## Methodology

The UN/SCN received 948 reports of nutrition surveys between October 1993 and April 2004 from 34 countries [15,16]. Of these, 17 countries were selected and all of the survey reports in these countries were reviewed for analysis. Survey reports were evaluated for 1) Validity of sampling methodology; 2) Precision of estimates; 3) Quality of measurements; and 4) Calculation of the prevalence of acute malnutrition and mortality rates. Only surveys conducted using random sample or exhaustive sample (i.e. including everyone in the population being studied) methodologies were included in the analysis. Reports that used convenience (e.g. non-random) sampling or did not measure the weight-for-height index for acute malnutrition were excluded. If information about one of the criteria mentioned above was not available from the survey report, the survey was classified as unknown for this criterion.

### Validity of sampling methodology

We classified surveys as valid, defined as the extent to which a variable measures what it is intended to measure, if they met the following criteria: 1) Random or systematic selection of households and/or children [8]; 2) For cluster surveys, ≥ 25 clusters were chosen using proportional-to-population-size sampling (PPS) and were actually surveyed [17,13]; 3) For cluster surveys, the selection of households during the second stage of sampling was undertaken by choosing one direction and selecting households by proximity or by systematic selection; 4) Inclusion of all children within the household or random selection of one child; 5) For exhaustive surveys, children had to have been measured in the household; those surveys that requested all children to gather at a central location were considered invalid; and 6) For morality surveys, all households, including those without children less than five years old, must have been included in the sampling [8].

### Precision of the prevalence of acute malnutrition and mortality rates

For nutrition surveys, the sample size needed depends on the estimated prevalence, design effect, precision, and level of confidence desired; the recall period is an additional factor for mortality surveys. Most guidelines on nutrition survey methodology recommend the use of a sample size of 450 children in systematic and random sampling and 900 children in cluster sample surveys, assuming a design effect of 2.0 [8,9]. Surveys were considered sufficiently precise if the sample size was large enough to allow the width of the 95% confidence interval to be within 30% of the estimated prevalence of acute malnutrition and within 50% of the estimated mortality rate. Since many surveys did not specifically state how they calculated their sample size, we used the estimated rate to re-calculate the sample size that would have been sufficient to allow for the desired precision [18] and compared this with the actual sample size. For surveys using cluster sampling, a design effect of 2.0 was assumed. Since confidence intervals and precision are not applicable for exhaustive surveys, if exhaustive surveys met the other criteria, we considered the precision and confidence intervals as acceptably meeting these criteria.

### Acute malnutrition: measurements, definitions and calculations

Measurements for acute malnutrition were valid if: 1) The children's age was between six and 59 months and/or height was used as a proxy for age and was between 65 and 110 cm; 2) Weight was measured with a precision of 100 g; 3) Height was measured with a precision of 1 mm; 4) Children less than 24 months or 85 cm were measured while lying down; and 5) oedema was included and measured correctly (i.e. if pitting remained after pressure was applied on both feet for at least 3 seconds). Survey measurements, definition of acute malnutrition and calculation of the prevalence of acute malnutrition were considered valid only if oedema had been measured correctly, included in the definition of acute malnutrition, and considered as an indicator of severe acute malnutrition. Acute malnutrition was defined as weight-for-height < -2 Z-scores of the NCHS/WHO reference value [19] (wasting) and/or oedema, and severe acute malnutrition was defined as weight-for-height < -3 Z-scores of the NCHS/WHO reference value (severe wasting) and/or oedema. If the results by category of nutritional status (i.e. number of children < - 3 Z-scores; number of children -2 to -3 Z-scores; number of children > -2 Z-scores; and number of children with oedema) were provided in the report, the prevalence of acute malnutrition was re-calculated. Data on the results provided in the nutrition survey reports were considered adequate if: 1) Prevalence of acute malnutrition and severe acute malnutrition were stated and correctly calculated; 2) Confidence intervals were reported; 3) Percentage of oedematous children was provided; 4) There was no misinterpretation of the results, such as disaggregating results by cluster or incorrect aggregation of several survey results together. Since the actual survey data were not available, it was not possible to check if the confidence intervals had been calculated properly according to the design of the survey methodology.

### Assessment of measles vaccination coverage

It is essential to measure measles vaccination coverage in humanitarian emergencies, as measles epidemics may lead to high numbers of deaths among children [20,21]. The current recommendation is to vaccinate children for measles from six months to 15 years in an emergency, and to repeat the vaccination at nine months for those children that had been vaccinated before nine months [6]. Since children between six and 59 months are already being surveyed for acute malnutrition, the assessment of measles vaccination coverage is easy to do and is recommended when nutrition surveys are undertaken [13]. In the surveys reviewed here, measles vaccination coverage was measured by examining cards and/or history of vaccination by guardian.

### Record of information on mortality and calculation of mortality rates

The survey reports were examined for the methodology used to obtain data on mortality. Both the current census approach (i.e. records the age of each person living in the household on the day of the survey, and the number of deaths and births within the household during the recall period) and the past census approach (i.e. records the age of each person living in the household at beginning of recall period, the number of births within the household during the recall period and the current status of these individuals) were considered acceptable [7]. When sufficient information was provided in the survey reports, mortality rates were re-calculated. Data on the results provided in the mortality survey reports were considered adequate if: 1) Mortality rates were correctly calculated; 2) Confidence intervals were reported; 3) Mortality rates were expressed as number of deaths per 10,000 per day. For this paper, we have primarily concentrated on the reporting of crude mortality rates (CMRs).

### Trends

The Cochran Armitage trend test [22,23] was applied for trend analysis on the proportion of nutrition and mortality surveys that met criteria for sampling validity, precision, measurement and calculation over the years.

## Results

Three hundred and sixty eight (368) survey reports conducted by 33 NGOs and international agencies in 17 countries (Afghanistan, Algeria, Angola, Bangladesh, Burundi, Central African Republic, Indonesia, Ivory Coast, Eritrea, Guinea, Mauritania, Nepal, Pakistan, Republic of Congo, Sri Lanka, Sudan, and Zambia) between October 1993 and April 2004 were evaluated (table 1).

**Table 1:** Number of surveys received by the UN/SCN from October 1993 to April 2004 by country and agency

Country	Number of nutrition surveys	Number of mortality surveys	Number of different agencies
Afghanistan	35	20	9
Algeria	2	0	2
Angola	70	48	12
Bangladesh	4	0	2
Burundi	49	29	8
Central African Republic	1	1	1
Ivory Coast	5	1	3
Eritrea	14	4	4
Guinea	13	2	3
Indonesia	4	0	2
Mauritania	1	1	1
Nepal	5	0	2
Pakistan	18	6	4
Republic of Congo	2	1	2
Sri Lanka	7	0	4
Sudan	134	80	17
Zambia	4	0	2
**Total**	**368**	**193**	

### Nutrition surveys

Criteria for sampling validity were met for 316 (85.9%) of the 368 surveys (table 2). The sample size was sufficient to allow for the width of the 95% CI to be within 30% of the estimated prevalence of acute malnutrition in 317 (86.1%) of the 368 surveys (table 2). All of the random sample surveys that used sample sizes of 450 children for random and systematic sampling (N = 14 of 26 surveys) and 900 children for cluster sampling (N = 167 of 300 surveys) were sufficiently precise. However, while those surveys using random and systematic sampling with fewer than 450 children were still sufficiently precise, 40% (N = 40 of 100 surveys) of cluster surveys that sampled < 900 children were insufficiently precise; half of these cluster surveys were conducted in Sudan with many of them occurring in 1999.

**Table 2:** Classification of 368 nutrition surveys from October 1993 to April 2004

	Total N (%)	Acceptable N (%)	Not acceptable N (%)	Unknown N (%)
**Sampling validity**				
Cluster	300 (81.5)			
First-stage PPS		268 (89.3)	2 (0.7)	30 (10.0)
No of clusters ≥ 25		290 (96.7)	3 (1.0)	7 (2.3)
PPS AND number of clusters ≥ 25		261 (87)	5 (1.7)	34 (11.3)
Systematic and random sampling	26 (7.0)			
Selection of households/children		22 (84.6)	0	4 (15.4)
Exhaustive surveys	35 (9.5)			
Survey conducted at household level		33 (94.3)	2 (5.7)	0
Unknown	7 (1.9)			7 (100)
**Met Sampling validity Criteria**	**368**	**316 (85.9)**	**7 (1.9)**	**45 (12.2)**
				
**Precision**				
Cluster	300	256 (85.3)	40 (13.3)	4 (1.4)
Random and systematic sampling	26	26 (100)		
Exhaustive surveys*	35	35 (100)		
Unknown	7			7 (1.9)
**Met Precision Criteria**	**368**	**317 (86.1)**	**40 (10.9)**	**11 (3.0)**
				
**Quality of measurements**				
Age/height range of inclusion (6–59 Months/65–110 cm)		320 (87.0)	15 (4.1)	33 (9.0)
Correct measurement of oedema		216 (58.7)	16 (4.3)	136 (40.0)
Correct measurement of weight and height		248 (67.4)	2 (0.5)	118 (32.1)
**Met Quality of Measurement Criteria**	**368**	**210 (57.1)**	**24 (6.5)**	**134 (36.4)**
				
**Definition of acute malnutrition**				
Standard definition of wasting		354 (96.2)	2 (0.5)	12 (3.3)
Inclusion of oedema as severe acute malnutrition		308 (83.7)	31 (8.4)	29 (7.9)
Prevalence expressed as Z-scores		343 (93.2)	25 (6.8)	
**Met Definition Criteria**	**368**	**295 (80.2)**	**42 (11.4)**	**31 (8.4)**
				
**Calculation of prevalence of acute malnutrition**				
Correct calculation of prevalence of acute malnutrition		165 (44.8)	8 (2.2)	195 (53.0)
CI given*		338 (91.8)	30 (8.2)	
% of oedema given		280 (76.1)	88 (23.9)	
Interpretation of results		311 (84.5)	57 (15.5)	
**Met Calculation Criteria**	**368**	**156 (42.4)**	**125 (34.0)**	**87 (23.6)**
				
**Sampling validity + Precision**				
Cluster	300	227 (75.7)	38 (12.7)	35 (11.6)
Systematic and random sampling	26	22 (84.6)	0	4 (15.4)
Exhaustive	35	33 (94.3)	2 (5.7)	0
Unknown	7	0	0	7 (100%)
**Met Sampling validity + Precision Criteria**	**368**	**282 (76.6)**	**40 (10.9)**	**46 (12.5)**
**Met Sampling validity + Precision + Quality of Measurement Criteria**	**368**	**189 (51.3)**	**60 (16.3)**	**119 (32.3)**
**Met Sampling validity + Precision + Quality of Measurement + Definition + Calculation Criteria**	**368**	**130 (35.3)**	**159 (43.2)**	**79(21.5)**

* Exhaustive surveys do not require precision or confidence intervals because they have included all persons in the population. We have included them as acceptable with regard to meeting the criteria

The vast majority of surveys (87.0%) correctly included children aged six to 59 months or 65 to 110 cm. The measurements met the quality criterion in 210 (57.1%) of the 368 surveys, the remainder being mainly due to missing information. Most of the surveys reported the prevalence of acute malnutrition in Z-scores (93.2%) and used a standard definition of wasting (96.2%). Thirty-one (8.4%) surveys did not include oedema in the definition of acute malnutrition, and it was not detected in 29 (7.9%) of the surveys. Overall, 295 (80.2%) of the 368 surveys met the criteria for definition of acute malnutrition.

Two-hundred and eighty (76.1%) of the surveys provided the percentage of oedematous children. Incorrect interpretation of results, as stated in Methods section, occurred in 57 (15.5%) of the survey reports. Of the 173 (47.0%) reports that allowed for verification of the calculation of the prevalence of acute malnutrition using weight-for-height and the presence of oedema, eight (4.6%) of the surveys, by four different agencies, had incorrectly calculated the prevalence; the proportion of surveys with incorrect prevalence calculations varied from 33% to 100%, depending on the agency. Overall, 156 (42.4%) of the 368 surveys met the criteria for correctly calculating the prevalence of acute malnutrition.

Two-hundred and eighty two (76.6%) of the surveys were both valid and precise. One-hundred and eighty-nine (51.3%) were valid, precise and met the quality of measurement criteria. Finally, 130 (35.3%) were valid and sufficiently precise, met the criteria for quality of measurement, outcome definition and calculation, while 159 (43.2%) did not satisfy all these quality criteria (table 2). The proportion of surveys meeting all criteria, as well as the proportion not meeting the quality criteria and the proportion classified as unknown varied significantly depending on the agency (chi-square = 83.7, p < 0.0001) (table 3).

**Table 3:** Classification of 368 nutrition surveys and 158 crude mortality rate (CMR) surveys from October 1993 to April 2004 by agency

**Agencies**	**Nutrition surveys**				**CMR surveys**			
	Total N	Acceptable N (%)	Not acceptable N (%)	Unknown N (%)	Total N	Acceptable N (%)	Not acceptable N (%)	Unknown N (%)

1	20	19 (95.0)	0	1 (5.0)	20	3 (15.0)	17 (85.0)	0
2	73	54 (74.0)	7 (9.6)	12 (16.4)	22	0	22 (100)	0
3	40	36 (90.0)	1 (2.5)	3 (7.5)	27	2 (7.4)	24 (88.9)	1 (3.7)
4	1	0	1 (100)	0	0	-	-	-
5	2	0	2 (100)	0	0	-	-	-
6	6	2 (33.3)	4 (66.7)	0	1	0	1 (100)	0
7	2	0	2 (100)	0	0	-	-	-
8	1	0	1 (100)	0	0	-	-	-
9	14	1 (7.1)	7 (50.0)	6 (42.9)	4	0	4 (100)	0
10	1	0	0	1 (100)	0	-	-	-
11	3	3 (100)	0	0	3	0	3 (100)	0
12	2	0	2 (100)	0	0	-	-	-
13	7	0	5 (71.4)	2 (28.6)	4	0	1 (25.0)	3 (75.0)
14	9	0	5 (55.6)	4 (44.4)	6	0	6 (100)	0
15	4	2 (50.0)	1 (25.0)	1 (25.0)	0	-	-	-
16	1	0	1 (100)	0	0	-	-	-
17	2	0	1 (50)	1 (50)	1	0	0	1 (100)
18	2	0	2 (100)	0	0	-	-	-
19	2	0	2 (100)	0	1	0	1 (100)	0
20	7	0	3 (42.9)	4 (57.1)	2	0	1 (50.0)	1 (50.0)
21	2	0	1 (50.0)	1 (50.0)	2	0	1 (50.0)	1 (50.0)
22	33	3 (9.1)	15 (45.4)	15 (45.4)	24	0	11 (45.8)	13 (54.2)
23	6	1 (16.7)	4 (66.7)	1 (16.7)	1	0	1 (100)	0
24	30	1 (3.3)	17 (56.7)	12 (40.0)	13	0	7 (53.8)	6 (46.1)
25	3	1 (33.3)	2 (66.7)	0	2	0	2 (100)	0
26	10	2 (20.0)	6 (60.0)	2 (20.0)	5	0	5 (100)	0
27	20	0	14 (70.0)	6 (30.0)	12	0	12 (100)	0
28	5	2 (40.0)	3 (60.0)	0	3	0	3 (100)	0
29	36	3 (8.3)	29 (80.6)	4 (11.1)	0	-	-	-
30	6	0	4 (66.7)	2 (33.3)	3	0	2 (66.7)	1 (33.3)
31	1	0	1 (100)	0	0	-	-	-
32	2	0	2 (100)	0	0	-	-	-
33	15	0	14 (93.3)	1 (6.7)	2	0	2 (100)	0

The prevalence of acute malnutrition ranged from 1.0% to 80.3% with a mean of 12.6%, a standard deviation of 9.3%, and a median of 10.1% (N = 368). The prevalence of severe acute malnutrition ranged from 0% to 48.5% with a mean of 2.2%, a standard deviation of 3.6%, and a median of 1.3% (N = 368). When only the 130 surveys that met all criteria were taken into account, the prevalence of acute malnutrition ranged from 1.0% to 45.5% with a mean of 11.4%, a standard deviation of 8.4%, and a median of 8.3%. The prevalence of severe acute malnutrition ranged from 0% to 20.9% with a mean of 2.0%, a standard deviation of 2.6%, and a median of 1.2%.

### Measles vaccination Coverage

Measles vaccination coverage could be assessed in 210 (57.1%) of the 368 surveys. Most of the surveys (72.3%) used card examination and history of vaccination; the same percentage of surveys reported measles vaccination among the nine to 59 month age group. The measles vaccination coverage range was from 0–100% with a mean of 65.1%, a standard deviation of 24.6%, and a median of 72.3%. When only surveys that met the criteria were taken into account, the measles vaccination coverage range was from 0–100% with a mean of 65.3%, a standard deviation of 27.4%, and a median of 72.4% (N = 134).

### Mortality surveys

Of the 368 nutrition reports screened, 193 (52.4%) nutrition surveys had an associated mortality survey. One hundred and fifty seven surveys (81.3%) assessed CMRs and under-five mortality rates (U5MRs), while 35 surveys (18.1%) only assessed U5MR and 1 survey (0.5%) only assessed CMR.

Among the 158 surveys that assessed CMRs, 87 (55.1%) met criteria for sampling validity (table 4). The precision met the criteria in 87 (55.1%) of the 158 CMR surveys and 60 (32.8%) of the 157 surveys having also assessed U5MR. Recall periods varied between one and 12 months with a median of three months, a mean of 4.1 months and a standard deviation of 3.3 months.

**Table 4:** Classification of 158 crude mortality rate (CMR) surveys from October 1993 to April 2004

	Total N (%)	Acceptable N (%)	Not acceptable N (%)	Unknown N (%)
**Sampling validity**				
Cluster	144 (91.1)			
First-stage PPS		135 (93.7)	2 (1.4)	7 (4.9)
No of clusters ≥ 25		142 (98.6)	2 (1.4)	0
PPS AND number of clusters ≥25		135 (93.7)	2 (1.4)	7 (4.9)
Systematic and random sampling	7 (4.4)			
Selection of households/children		7 (100)		
Exhaustive surveys	7 (4.4)			
Survey conducted at household level		7 (100)		
Unknown	0			
All households included, regardless of the presence of under-five children	158	88 (55.7)	13 (8.2)	57 (36.1)
**Met Sampling validity Criteria**	**158**	**87 (55.1)**	**15 (9.5)**	**56 (35.4)**
				
**Precision**				
Cluster	144	80 (55.5)	27 (18.7)	37 (25.7)
Random and systematic sampling	7	1 (14.3)	0	6 (85.7)
Exhaustive surveys*	7	7 (100)		
Unknown	0			
**Met Precision Criteria**		**88 (55.7)**	**27 (17.1)**	**43 (27.2)**
				
**Calculation of CMR**				
Correct calculation of mortality rate	158	43 (27.2)	9 (5.7)	106 (67.1)
CI given*	158	38 (24.1)	120 (75.9)	0
CMR expressed as deaths/10,000/day	158	158 (100)	0	0
**Met Calculation Criteria**	**158**	**7 (4.4)**	**120 (75.9)**	**31 (16.2)**
				
**Sampling validity + Precision**				
Cluster	144	54 (37.5)	35 (24.3)	55 (38.2)
Systematic and random sampling	7	1 (14.3)	3 (42.9)	3 (42.9)
Exhaustive	7	6 (85.7)	0	1 (14.3)
Unknown	**0**			
**Met Sampling validity + Precision Criteria**	**158**	**61 (38.6)**	**38 (24.1)**	**59 (37.3)**
**Met Sampling validity + Precision + Calculation Criteria**	**158**	**5 (3.2)**	**126 (79.7)**	**27 (17.1)**

* Exhaustive surveys do not require precision or confidence intervals because they have included all persons in the population. We have included them as acceptable with regard to meeting the criteria

Thirty-five (22.1%) of the 158 CMR surveys used the current census methodology; the methodology was unknown for the other surveys. Most surveys (93.0%) did not state if births were taken into account.

Of the 52 (32.9%) surveys from six agencies that allowed for the recalculation of the CMR, nine (17.3%) CMRs, all from one agency, had been incorrectly calculated due to simple mathematical errors (table 4). The proportion of miscalculations was 45% for this agency. Confidence intervals were provided for 38 (24.1%) of the 158 surveys. All survey results expressed number of deaths per 10,000 per day.

Sixty-one (38.6%) of the surveys were both valid and precise. Only five (3.2%) were valid, precise and met the calculation criteria (table 4). The proportion of surveys classified as acceptable, not acceptable and unknown varied significantly depending on the agency (table 3).

The CMR ranged from 0.5 to 10.0 deaths per 10,000 persons per day with a mean of 2.02, a standard deviation of 2.09, and a median of 1.21 (N = 158). The U5MR ranged from 0.38 to 25.0 deaths per 10,000 per day with a mean of 3.56, a standard deviation of 3.91, and a median of 2.45 (N = 192).

### Trend analysis

**Figure 1:**
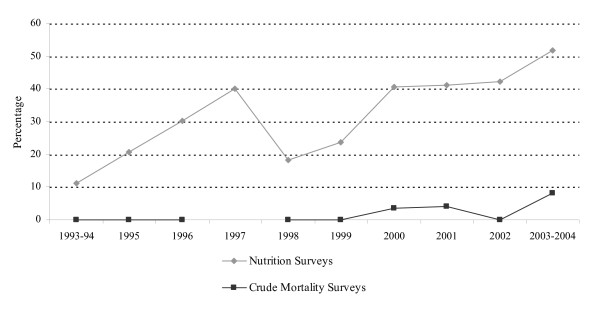
Trend in proportion of 368 nutrition and 158 crude mortality rate surveys which met criteria for sampling validity, precision, quality of measurements and calculation from October 1993 to April 2004.

The proportion of nutrition surveys that met criteria for sampling validity, precision, measurement, definition and calculation rose significantly from 11.1% in 1993–94 to 51.7% in 2003–2004 (p-value for trend < 0.0001) (figure 1).

The implementation of CMR surveys associated with nutrition surveys increased significantly over the years (p-value for trend < 0.0001) (table 5), but the proportion of CMR surveys that met criteria for sampling validity, precision and calculation did not differ (p-value for trend = 0.165) (figure 1).

**Table 5:** Number of nutrition surveys and crude mortality rate (CMR) surveys by year, according to survey reports received by the UN/SCN between October 1993 and April 2004 from 17 countries

Year	Number of nutrition surveys	Nutrition surveys that included CMR surveys n (%)
1993–94	27	3 (11.1)
1995	29	3 (10.3)
1996	23	4 (17.4)
1997	20	0 (0.0)
1998	22	9 (40.9)
1999	38	16 (42.1)
2000	32	28 (87.5)
2001	34	24 (70.6)
2002	83	35 (42.2)
2003–04	60	36 (60.0)

## Discussion

Nutrition and mortality data in humanitarian emergencies are the most widely accepted indicators for assessing the degree of a crisis and with which to make decisions [6,7]. Good quality data that are valid and sufficiently precise are necessary for decision-making. Programmatic and funding decisions rely on such data; funding decisions from a limited amount of resources are made in part due to these data. Previous surveys in Somalia, Iraq and Ethiopia have shown that there were significant deficiencies in the sampling validity and precision of nutrition [12-14] and mortality surveys [12] in humanitarian emergencies. Our paper shows similar deficiencies but better compliance with agreed upon international standards than the other studies. When the results of all surveys are compared with those that met all criteria, it is clear that those surveys that did not meet all criteria had many outliers, which increased the range and median of the overall results. Furthermore, the large number of surveys examined over a 10-year period showed that the proportion of nutrition surveys that met the criteria for sampling validity, precision, measurement, definition and calculation rose significantly from 1993/94 to 2003/04. Although the number of CMR surveys associated with nutrition surveys increased significantly over time, the proportion of these surveys that met the above criteria did not. Some errors were more commonly made than others (table 6). Correcting them would lead to a significant improvement of the quality of the surveys.

**Table 6:** Most common errors in nutrition and mortality surveys, and recommendations for improvement

**Most common errors**	**Recommendations**
Measurement techniques for weight, height, oedema and recording of mortality not mentioned in reports.	Include in the report details on the techniques used for measurements and recording of mortality.
Oedema not measured and/or not correctly taken into account in calculation of malnutrition prevalence.	Always measure oedema and ensure that oedematous children are correctly included in calculation of prevalence of malnutrition.
Insufficient precision of survey results.	Pay attention to the recommendations used to calculate sample sizes and their precision for malnutrition and mortality surveys. Calculate sample sizes for malnutrition and mortality surveys independently.
Confidence intervals of mortality rates not calculated.	Calculate accurate confidence intervals for mortality rates with user-friendly software that takes into account design effect when cluster sampling is used.
Incorrect interpretation of results.	Do not aggregate several survey results together without weighing; do not disaggregate cluster surveys according to clusters/certain aggregates of clusters.

Sampling methodology was valid in most of the nutrition surveys indicating that guidelines were being appropriately followed. However, only slightly more than half of the mortality surveys were considered valid, mainly due to a lack of specificity in the reports as to how household selection occurred; this is crucial for the representativeness of the surveys, to ensure that all households were included, regardless of the presence of children aged six to 59 months.

The precision criteria chosen for this analysis are expressed as a percentage of the observed estimate of acute malnutrition prevalence and mortality rate. Therefore, the lower the estimates, the higher the sample size required will be. This may mean that, in certain circumstances, required sample sizes may be large and thus, the implementation of the survey may require more time, personnel and funds. However, for most surveys, such precision is necessary to allow for prioritisation of programme implementation, as well as to provide a baseline with sufficient precision to adequately monitor future trends and, therefore, programme effectiveness.

Most nutrition surveys were sufficiently precise according to our criteria. However, there was a lack of precision of nutrition surveys analysed in Sudan in 1999. This was likely due to the use of the same sample sizes and thus absolute precision (e.g. ± 5% around the estimated prevalence) as that used in the 1998 surveys – when the prevalence of acute malnutrition was extremely high in parts of Sudan – as opposed to basing sample size on relative precision (e.g. within 30% of the estimated prevalence). For example, if the acute malnutrition of a population is 35% then a precision of ± 5% (that is, a 95% confidence interval of 30%–40%) may be acceptable, whereas this would not be the case if the prevalence is 10% (a 95% confidence interval of 5%–15%). Most nutrition guidelines recommend that a sample size of 900 children in cluster sampling and 450 children in systematic or random sampling be used [8,9]. If this had been applied in all surveys we analysed, they would have had a sufficient sample size to achieve an adequate level of precision. However, some surveys would still have been sufficiently precise with a lower sample size. The Standardize Monitoring and Assessment of Relief and Transitions (SMART) manual recommends that sample size be calculated rather than a fixed sample size of 900/450 children used, assuming that in many situations a lower sample size may be sufficient and this would make surveys logistically simpler and possibly reduce bias [7]. Our analysis shows, however, that the recommendation of a specific sample size is a safeguard, especially useful when no recent data on the prevalence of acute malnutrition in the area exist or when staff is insufficiently trained to properly estimate the appropriate sample size.

Just over half of the CMR surveys were sufficiently precise. This was likely because the sample sizes for mortality surveys often used the same sample sizes calculated for nutrition surveys. Sample sizes for each key variable in a survey must be calculated independently. In the case of mortality surveys, expected mortality rate, recall period and precision required need to be considered. Recently some guidelines for the calculation of sample sizes for mortality surveys and software for their calculation have become available [7,18,24].

Many reports lacked structured methodology and results sections and had insufficient detail to allow for proper analysis and evaluation. For example, there were insufficient descriptions of the measurements of malnutrition and mortality in the surveys evaluated (about 40% and 80%, respectively). For nutrition surveys, the percentage of oedematous children was not reported in approximately 25% of the reports. Although this is better than found in another review [12], proper assessment and reporting of oedematous children need to be strengthened. Almost all surveys in our study reported acute malnutrition prevalence in Z-scores, the recommended method [9]; this is higher than in previous studies [12,13]. The proportion of mortality surveys which mentioned if births were taken into account was very low (1.8%) and similar to the Boss et al. study, in which no reports mentioned births [12].

The quality of calculations depended upon the software used for survey analysis. Among the limited number of surveys for which the calculations of acute malnutrition prevalence could be checked (N = 173), approximately 33% of the calculations performed with EpiInfo 6 were incorrect, because oedematous children had not been correctly taken into account; they had either been counted twice (i.e. once as oedematous children and once according to their weight-for-height index) or they had only been included in the calculation according to their weight-for-height index [25]. Conversely, all such calculations performed with EpiInfo 5/Epinut 2 were correct. EpiInfo 5/Epinut 2 automatically provides the calculation and 95% confidence intervals of acute and severe acute malnutrition while taking into account oedematous children as severely malnourished. In contrast, with the Epinut version of EpiInfo 6, users need to go through a cumbersome and non-intuitive process to calculate the prevalence of acute malnutrition and 95% confidence interval that properly includes oedematous children. No comprehensive guideline to analyse nutrition surveys using EpiInfo 6 was available before 2004, when a manual that extensively describes the procedure was released [26]. Until recently, there was also no software especially designed for the calculation of mortality rates and confidence intervals [7,18]. This new software allows for the analysis of nutrition and mortality surveys that includes a set of indicators to aid in the assessment of the methodological quality and appropriate measurements performed during a survey.

Only approximately 50% of the surveys assessed measles vaccination coverage. Although our results were higher than in another review [13], this is still unacceptable. Furthermore, the mean and median of measles vaccination coverage were below the recommended 90% [6], suggesting that measles epidemics are possible in many populations surveyed. As recommended previously, all nutrition surveys among children should include a measles vaccination coverage component and should clearly disaggregate in the report the percentage according to both history and vaccination card as well as card alone.

There are some limitations in this paper. Although the nutrition surveys analysed were randomly selected from the global database, selection bias is a possibility. However, there was no difference between the global database and the sample selected regarding the distribution of nutrition survey reports by year (χ^2 ^= 12.6, p = 0.18), or by agency (χ^2 ^= 15.3, p = 0.06). Furthermore, the reports analysed are sent to the UN/SCN on a voluntary basis and it is possible that the surveys included in this study do not represent all the surveys that were conducted at country level. Although the methods for second-stage sampling were not always stated in the reports and not systematically recorded in our study, some surveys chose only one child per household. If appropriate weighing was not performed during the analysis, this might have introduced a bias.

In our review, the precision of nutrition and mortality surveys was analysed assuming a design effect of 2.0 for cluster-sampled nutrition and mortality surveys. Recent research shows that a design effect of some nutrition and non war-related mortality surveys was below 2.0 and closer to 1.5 [27]. If a design effect of 1.5 had been used in our analysis, the proportion of nutrition and mortality surveys for which the sample size was sufficient would have been higher. Conversely, war-related mortality surveys might have a higher design effect and might require a higher sample size [27]. In this case, some of the surveys that were classified as sufficiently precise may not have been.

The analysis was performed according to the information available in the survey reports. Where information was lacking, criteria were classified as not being met. Thus, misclassification could have occurred, especially among those surveys that did not report on measurement procedures of weight, height and oedema and on how mortality rates were calculated; this may have caused an underestimation of those surveys that met certain criteria. Adequate implementation of the surveys at field level, such as the application of the sampling methodology and the quality of measurements could not be verified, nor could primary data analysis be undertaken, as the actual datasets were unavailable to us.

Survey reports did not clearly define a decision matrix for the recording of measles vaccination coverage (e.g. if vaccination card available, this result is used; if not available, then oral history is used). Therefore, although this would appear to be the logical decision matrix, there may be some surveys that reported the results differently.

Several factors are essential to the strengthening of the quality of nutrition and mortality surveys. Appropriate guidelines are now available and need to be widely disseminated; the increased availability and access to the internet in remote areas has improved their distribution. However, the most crucial factor is the availability of well-trained and experienced staff. This should also reduce the incorrect interpretation of results that were noted in our analysis. Unfortunately, the high turnover of personnel working in emergency situations leads to the loss of experienced people. The proportion of surveys that did not meet the criteria varied significantly depending on the agency. This supports results from a recent article [28] and shows that there is the need for systematic and repetitive training and capacity building at field, national and international levels. In many circumstances a specialised agency or a consultant with technical expertise and experience are needed to competently undertake and make a sufficiently detailed report for distribution.

Initiatives aiming to improve and standardise survey methodology have been initiated. In some countries, coordination bodies led by different agencies in collaboration with national ministries have been implemented. For example, the Food and Agricultural Organization, through the Food Security Analysis Unit, coordinates nutrition surveys and their analysis together with the food security situation and maintains a nutrition survey database in Somalia since 1994; an Emergency Nutrition Coordination Unit was established within the Ethiopian government in 2000 to coordinate nutrition surveys, provide training according to national guidelines and analyse the nutrition situation; UNICEF established a nutrition surveillance system with a publication of bi-monthly nutrition updates in mid-2005 in Darfur. Furthermore, an international inter-agency initiative was launched in 2002 to SMART. The first version of a guideline on how to undertake nutrition and mortality surveys together with software to analyse them have recently been produced [7,18]. This initiative, led by USAID, reflects a tendency that has developed over recent years by donors to establish a range of standards and indicators to inform the planning, coordination, monitoring and evaluation of humanitarian response. A health and nutrition tracking service as part of the humanitarian reform process for the nutrition and health clusters is in development for populations of humanitarian concern. Although these are important initiatives, there is a need to strengthen and systematise training and coordination at country and international levels.

There are still a number of methodological issues in nutrition and mortality surveys in humanitarian emergencies that need further study. These include, but are not limited to, the mapping of the affected populations for first stage cluster sampling and the selection of households for the second stage, documenting different design effects among various outcomes in different situations, and different methodologies to record deaths, births, and migration in mortality surveys. Furthermore, there is a need to consider standardised age distribution in order to compare across different situations for mortality surveys. The introduction of the recently released WHO growth standards [29] suggests important differences in the diagnosis of wasting compared to the NCHS growth reference [30,31] and will thus make comparison of trends in malnutrition in different regions more complicated. Although not analysed in this review, other aspects that need further examination beyond survey methodology include the delimitation of the area surveyed according to geographical boundaries or to livelihood zones and the timing of the surveys according to seasonal variations. Alternatives to the standard cluster or random sampling methodologies have also been proposed and need further investigation. Moreover, the prevalence of acute malnutrition and mortality rates alone are not sufficient to assess the situation. The examination of other related factors such as food security, access to health care and safe water, and protection are fundamental underlying issues that must be assessed in order to help explain the nutrition and mortality outcomes of the surveys. Finally, there is a need for a central and accessible site on the internet not only for survey reports, but also the actual data. This repository will help to ensure quality of surveys and aid in future research.

## Competing interests

The author(s) declare that they have no competing interests.

## Authors' contributions

CP was responsible for data collection and analysis, and drafted the article. PS contributed to the conception and design of the study, the interpretation of the results and co-wrote the paper. All authors read and approved the final manuscript.
